# The anti-inflammatory and anti-apoptotic effects of *Achillea millefolium* L. extracts on *Clostridioides difficile* ribotype 001 in human intestinal epithelial cells

**DOI:** 10.1186/s12906-024-04335-2

**Published:** 2024-01-13

**Authors:** Hamideh Raeisi, Masoumeh Azimirad, Samaneh Asadi-Sanam, Hamid Asadzadeh Aghdaei, Abbas Yadegar, Mohammad Reza Zali

**Affiliations:** 1 Foodborne and Waterborne Diseases Research Center, Research Institute for Gastroenterology and Liver Diseases, Shahid Beheshti University of Medical Sciences, Tehran, Iran; 2 Medicinal Plants Research Division, Research Institute of Forests and Rangelands, Agricultural Research, Education & Extension Organization (AREEO), Tehran, Iran; 3 Basic and Molecular Epidemiology of Gastrointestinal Disorders Research Center, Research Institute for Gastroenterology and Liver Diseases, Shahid Beheshti University of Medical Sciences, Tehran, Iran; 4 Gastroenterology and Liver Diseases Research Center, Research Institute for Gastroenterology and Liver Diseases, Shahid Beheshti University of Medical Sciences, Tehran, Iran

**Keywords:** *Achillea millefolium* L., Extracts, *Clostridioides difficile*, Ribotype 001, Tox-S, Inflammation, Apoptosis

## Abstract

**Background:**

*Clostridioides difficile* infection (CDI) is one of the most common health care-acquired infections. The dramatic increase in antimicrobial resistance of *C. difficile* isolates has led to growing demand to seek new alternative medicines against CDI. *Achillea millefolium* L. extracts exhibit strong biological activity to be considered as potential therapeutic agents. In this work, the inhibitory effects of *A. millefolium*, its decoction (DEC) and ethanol (ETOH) extracts, were investigated on the growth of *C. difficile* RT001 and its toxigenic cell-free supernatant (Tox-S) induced inflammation and apoptosis.

**Methods:**

Phytochemical analysis of extracts was performed by HPLC and GC analysis. The antimicrobial properties of extracts were evaluated against *C. difficile* RT001. Cell viability and cytotoxicity of Caco-2 and Vero cells treated with various concentrations of extracts and Tox-S were examined by MTT assay and microscopy, respectively. Anti-inflammatory and anti-apoptotic effects of extracts were assessed in Tox-S stimulated Caco-2 cells by RT-qPCR.

**Results:**

Analysis of the phytochemical profile of extracts revealed that the main component identified in both extracts was chlorogenic acid. Both extracts displayed significant antimicrobial activity against *C. difficile* RT001. Moreover, both extracts at concentration 50 µg/mL had no significant effect on cell viability compared to untreated cells. Pre-treatment of cells with extracts (50 µg/mL) significantly reduced the percentage of Vero cells rounding induced by Tox-S. Also, both pre-treatment and co-treatment of Tox-S stimulated Caco-2 cells with extracts significantly downregulated the gene expression level of IL-8, IL-1β, TNF-α, TGF-β, iNOS, Bax, caspase-9 and caspase-3 and upregulated the expression level of Bcl-2.

**Conclusion:**

The results of the present study for the first time demonstrate the antimicrobial activity and protective effects of *A. millefolium* extracts on inflammatory response and apoptosis induced by Tox-S from *C. difficile* RT001 clinical strain in vitro. Further research is needed to evaluate the potential application of *A. millefolium* extracts as supplementary medicine for CDI prevention and treatment in clinical setting.

**Supplementary Information:**

The online version contains supplementary material available at 10.1186/s12906-024-04335-2.

## Background

*Clostridioides* (formerly, *Clostridium*) *difficile* is a Gram-positive, spore-forming anaerobic bacterium, which is known as the most common cause of healthcare-associated pathogen. *C. difficile* infection (CDI) can lead to a range of different diseases in humans such as asymptomatic colonization, antibiotic-associated diarrhea (AAD), potentially pseudomembranous colitis (PMC), and toxic megacolon [1, 2]. In recent decades, the incidence of CDI has been increasing in both adult and pediatric populations, and the surveillance data from 2011 estimated the number of CDI about 453,000 cases with nearly 29,000 deaths in the United States [3, 4]. Typically, the disturbance of the normal gut microbiota during or after broad-spectrum antibiotic treatment could be regarded as a major risk factor associated with the development of CDI [4, 5]. The main symptoms of CDI are correlated with the production of two major toxins, toxin A (TcdA) and toxin B (TcdB), which trigger cytopathic effect (CPE) in intestinal epithelial cells (IECs) through glycosylation and inactivation of Rho/Ras proteins [6]. These events can lead to cytoskeleton disintegration, and ultimately loss of epithelial barrier function, and apoptosis [7]. Additionally, the secretion of these toxins into the gastrointestinal tract provokes intracellular signaling cascades, which result in induction of severe inflammation and eventually cell death [8]. These outcomes damage the patient’s colonic mucosa and cause severe diarrhea.

The conventional treatment recommended for mild-to-moderate CDI includes antibiotic therapy, particularly vancomycin or metronidazole, which are non-selective, thus consequently leading to further irritating gut dysbiosis (imbalance of gut microbiota) and reduction of the normal gut commensals [4, 9]. The gut dysbiosis leads to favoring an appropriate niche for antibiotic-resistant strains of *C. difficile* and facilitates their intestinal colonization [10, 11]. Alternatively, administration of recommended anti-CDI antibiotics is associated with a high recurrence rate (20–40%) of CDI (rCDI) in patients with primary infection within 4 to 6 weeks after completion of antibiotic therapy [11]. Recently, the use of specific anti-*C. difficile* antibiotics, such as fidaxomicin, has been suggested as an efficient option for reduction of the relapse rate of CDI, however, the high costs of fidaxomicin restrict its widespread application [12, 13].

Currently, to avoid the detriments of antibiotics, alternative therapeutic approaches have been introduced for the treatment or prevention of CDI, including fecal microbiota transplantation (FMT) and antibody-based immunotherapy [14, 15]. Additionally, pharmacological actions of plant-derived compounds have attracted much attention due to their antimicrobial, antioxidant, and anti-inflammatory properties in the last decades [16–19]. The anti-inflammatory properties of components extracted from medicinal herbs have been reported for modulating the severity of inflammatory bowel disease (IBD) in both in vitro and in vivo models [16, 20]. Notably, the anti-inflammatory properties of plant products have been associated with the modulation of oxidative stress in intestinal cells [18, 21] and reinforce the function of the epithelium barrier [22]. Moreover, herbal-derived compounds exert antimicrobial activity and can modulate and attenuate the inflammatory responses induced by pathogenic microbes, thus, may be highly attractive as potential complementary therapies [23].

Among the most studied plant-derived components, *Achillea millefolium* L. extracts have been frequently studied in recent years [18, 24]. *Achillea* decoction is a widely used traditional medicine for the treatment of a variety of gastrointestinal diseases in several regions of the world [24–26]. Additionally, maceration in different solvents is a simple extraction method to prepare bioactive compounds from the plants [27]. Several in vitro and in vivo studies have reported a wide array of biological features for *A. millefolium* and its derivatives, including antimicrobial [24, 28], antioxidant and anti-inflammatory properties [18, 29], and gastro-protective activities [30] in its aqueous, hydroalcoholic, and methanol extracts. Moreover, the anticancer activity of *A. millefolium* extracts is demonstrated in various tumor cell types, including cervical and breast epithelial adenocarcinoma, skin epidermoid carcinoma [31], hepatoma [32], and lung tumor cells [33].

In view of the potential biological efficacy of different extracts of *A. millefolium* on infectious diseases, we investigated the antimicrobial activities of decoction (DEC) and ethanol (ETOH) extracts of *A. millefolium* on a toxigenic *C. difficile* clinical strain ribotype (RT) 001 (RT001). Viability and cytotoxicity of Caco-2 and Vero cells treated with DEC, and ETOH extracts and the cell-free supernatant (Tox-S) of *C. difficile* RT001 strain were also examined. Additionally, the inhibitory effects of both extracts on Tox-S mediated cytotoxicity were determined in Vero cells. We also assessed the modulatory effects of these extracts on expression level of the genes involved in inflammation and apoptosis in Caco-2 cells stimulated by Tox-S.

## Materials and methods

### Plant materials and extract preparation

The plant materials were collected in March 2021 from various regions of Chaharmahal and Bakhtiari province, in southwestern of Iran. *A. millefolium* species was identified and verified by Dr. Vali Allah Mozaffarian at the Research Institute of Forest and Rangelands (AREEO, Tehran; Iran), and kept under code number: 353 in the herbarium for future reference. The vegetal materials consisting of *A. millefolium* flowers were washed with distilled water, dried, grind, and used for preparing ETOH and DEC extracts. For ETOH extract, 20 gr of powder was suspended in 200 mL of 70% ethanol (v/v), and macerated under continuous mechanical stirring for 24 h, followed by filtration through a paper filter. The DEC extract was prepared by boiling 20 gr of powder in distilled water for 30 min, and then incubated at room temperature for a further 30 min and passed through a paper filter. The obtained samples for both ETOH and DEC were centrifuged at 3000 × g for 15 min. The supernatants were isolated and filtered through 0.22 μm membrane filters (Sartorius, USA). The filtered extracts were evaporated to dryness at 60 °C using a rotary evaporator, then freeze-dried and ground into a powder. The herbal extracts were stored at -20 °C until further experiments.

### Determination of phytochemical compounds of plant extracts

The chemical analysis of *A. millefolium* extracts was performed by using high-performance liquid chromatography (HPLC) and gas chromatography (GC). In this regard, analysis of the phenolic compounds of DEC and ETOH extracts was performed by using an HPLC system (Agilent 1260 Infinity II Quat Pump, CA, USA) equipped with photodiode array detector (PDA WR detector, CA, USA). A high-pH stable Ultisil XB-C18, 5 μm column (column dimension: length × ID = 250 × 4.6 mm) was used for separation. The mobile phases used included solvent A (acetic acid:water, 1:99 v/v) and solvent B (methanol). The injection volume was 10 µL to detect eight compounds (gallic acid, catechin, rutin, quercetin, chlorogenic acid, caffeic acid, rosmarinic acid, and apigenin). Analyses were carried out at 30 °C with a flow rate of 1.0 mL/min. The analytes were monitored using a PDA detector at 254, 280, 300, and 330 nm. The concentration of eight expected phenolic components in both extracts was identified by comparing with retention times (Rt) and UV spectra of their respective standards (Sigma-Aldrich, Germany).

GC Analysis of volatile compounds of DEC and ETOH extracts was performed by using an Agilent 7890 (Agilent Technologies, USA) coupled with Flame Ionization Detector (FID) and a nonpolar DB-5 fused silica column (length 30 m, inner diameter 0.25 mm, and thickness of stationary phase layer equal to 0.25 μm). The initial column temperature was 60 °C and programmed to increase to 260 °C at a rate of 3 °C/min. The injector temperature was 260 °C and nitrogen was used as the carrier gas with a flow rate of 1.0 mL/min. The split ratio was 10:1. The amount of components including limonene, 1,8-cineole, and menthone in plant extracts was determined using the injection of standard samples to GC instrument and calibration curve.

### *C. difficile* strain and culture conditions

In this study, we used the toxigenic strain of *C. difficile* RT001 (A^+^B^+^), which was previously characterized in the Department of Anaerobic Bacteriology in Research Institute for Gastroenterology and Liver Diseases in Tehran, Iran [34]. The strain was cultured on cycloserine-cefoxitin-fructose agar (CCFA, Mast) supplemented with 5% (v/v) sheep blood under anaerobic conditions (85% N_2_, 10% CO_2_ and 5% H_2)_ (Anoxomat® Gas Exchange System, Mart Microbiology BV) at 37 °C for 48–72 h after an alcohol shock treatment [35, 36].

### Preparation of Tox-S

The Tox-S preparation was carried out as described previously [36]. In brief, the *C. difficile* RT001 was grown anaerobically in CCFA for 48 h and then used to prepare a bacterial suspension equal to 2 McFarland turbidity standard in 0.85% sterile saline. The suspension (100 µL) was transferred to a 10 mL pre-reduced brain heart infusion (BHI) broth and incubated at 37 °C under agitation at 120 rpm for 72 h. After that, cells and debris were removed by centrifugation at 4,000 × g in 4 °C for 5 min. The supernatant was aseptically filtered through a membrane filter with 0.22 μm pore size, and kept at -80 °C until analysis. The presence of *C. difficile* TcdA/B toxins in the supernatant was evaluated by enzyme-linked immunosorbent assay (ELISA, Generic Assays, Germany) according to the manufacturer’s instructions.

### Antimicrobial assays by minimum inhibitory concentration (MIC) determination

To determine the antibacterial activity of the plant extracts, agar dilution and broth microdilution methods were applied as previously described by Azimirad et al. [36] with minor modifications. Briefly, the range of concentrations used to determine MICs of the extracts was 5-200 µg/mL prepared in the 10% dimethyl sulfoxide (DMSO). Moreover, bacterial suspension from RT001 strain at a final turbidity of approximately 10^6^ CFU/mL was prepared from pure cultures recovered anaerobically on CCFA plates at 37 °C for 48 h. For agar dilution method, different concentrations of each extract were added in pre-reduced supplemented Brucella agar. Plates were inoculated with a final inoculum density of approximately 1 × 10^6^ CFU/mL from RT001 strain and inoculated under an anaerobic atmosphere at 37 °C for 48 h. MICs were determined as the lowest concentration of each extract at which no visual growth of bacteria was observed. Growth controls were performed by addition of bacterial inoculum into a free medium without extract. The plates without bacterial inoculation and plates containing 10% DMSO were served as negative controls. Three repeats were considered for each assay.

For broth microdilution, a 96-well microtiter plate was filled with 0.5 mL of sterilized pre-reduced Brucella broth supplemented with different concentrations of the extracts, 1 µg/mL vitamin K1, 1 µg/mL L-cysteine, 5 µg/mL hemin, and 5% (v/v) lacked sheep blood and then inoculated with approximately 10^5^ CFU/well of *C. difficile* RT001. The wells without any extracts served as bacterial growth control. The wells without bacterial addition and wells containing 10% DMSO were served as negative controls. Plates were then incubated under anaerobic conditions at 37 °C for 48 h. MICs were determined for the lowest concentration of each extract, where no visible growth was seen by evaluation of turbidity using optical density reading at 600 nm using a microplate reader (BioTek, USA). Moreover, percentage of growth inhibition for each extract against *C. difficile* was determined by comparing OD of each treatment to control. Three repeats were considered for each assay.

### Cell culture and growth conditions

The Caco-2 (human colon adenocarcinoma cell line) and Vero cells (an African green monkey kidney cell line) were obtained from the Iranian Biological Resource Center (IBRC) and cultured in high-glucose Dulbecco’s modified Eagle’s medium (H-DMEM) (Gibco, USA) supplemented with 10% (v/v) fetal bovine serum (FBS) (Gibco, USA), 1% (v/v) penicillin/streptomycin (Sigma-Aldrich, Germany), and 1% (v/v) of a non-essential amino acid (Gibco, USA).

### Cell viability assay

To evaluate cell viability, a colorimetric assay based on the cleavage of the yellow tetrazolium salt 3-(4,5 dimethylthiazol-2-yl)-2,5-diphenyl tetrazolium bromide (MTT) (Sigma-Aldrich, Germany) was performed as previously described [36]. MTT assay examines the metabolic activity of the cells by measuring the reduction of tetrazolium salts to colored formazan products. To do this, DEC and ETOH extracts and Tox-S were individually titrated on Caco-2 cells that were seeded at 5 × 10^3^ cells/well in 96-well plates and incubated for 1, 4, 24 h time points at 37 °C in a 5% CO_2_ incubator. At the end of incubation times, cells were washed twice with phosphate-buffered saline (PBS, pH 7.4) and then 90 µL of culture medium and 10 µL/well of MTT (5 mg/mL in PBS) was added to each well. After incubation of cells for 4 h at 37 °C under 5% CO_2_ atmosphere, 200 µL of DMSO was added to each well for 15 min to stop the reaction. Untreated monolayers were used as negative controls and wells without cells served as blanks. The plates were shaken and then incubated at 37 °C for 10 min. The absorbance was recorded at 570 nm (reference filter, 690 nm) by a microplate reader (BioTek, USA). The percentage of cell viability was calculated as follows: cell viability (%) = (absorbance of treated cells × 100%)/absorbance of untreated cells. All experiments were performed in triplicate.

### Cytotoxicity assay

The cytotoxic activity of the extracts and Tox-S was determined using Vero cells by breakdown of the actin cytoskeleton, which leads to cell rounding, as previously described [36, 37]. Briefly, Vero cells were seeded in a 96-well plate at 10^4^ cells/well. Two concentrations of *C. difficile* RT001 Tox-S (100 and 500 µg/mL) and two concentrations of DEC and ETOH extracts (50 and 75 µg/mL) were used in cytotoxicity assays. In addition, to determine the ability of the extracts to inhibit the cytotoxicity caused by Tox-S, each extract was combined at concentration 50 µg/mL with Tox-S at different consecrations (100 and 500 µg/mL), and were added to the Vero cells, and incubated for 4, 8, and 24 h at 37 °C in 5% CO_2_ conditions. The cytotoxic activity was determined by 90% cell rounding that was detected visually using an inverted microscope (Olympus Corporation, Tokyo, Japan) at ×200 magnification. Cell images were taken and the percentage of round cells in different treatments was determined by ImageJ software-assisted counting, and then normalized to the percentage of round cells in the wells treated with Tox-S. The assays were carried out in triplicate.

### Treatment of Caco-2 cells with Tox-S and *A. millefolium* extracts

Caco-2 cells were seeded into 24-well plates at a density of 2.5 × 10^4^ cells*/*well. Three forms of cell treatments were carried out as follow: (1) Caco-2 cells were treated with Tox-S (100 µg/mL), DEC, or ETOH extracts (50 µg/mL) and incubated for 4, 8, and 24 h at 37 °C in 5% CO2 atmosphere. (2) Caco-2 cells were pre-treated with extracts (50 µg/mL), and after 8 h of incubation, cells were exposed to Tox-S (100 µg/mL) and incubated for further 4 and 24 h at 37 °C in 5% CO_2_ atmosphere. (3) Caco-2 cells were simultaneously treated with the extracts (50 µg/mL) and Tox-S (100 µg/mL), and incubated for further 4 and 24 h at 37 °C in 5% CO_2_ conditions. Untreated cells and Tox-S (100 µg/mL) without the addition of each extract were used as controls. After treatments, the cells were lysed for RNA extraction and gene expression analysis. All treatments were run in triplicate.

### Total RNA isolation and cDNA synthesis

The treated Caco-2 cells were collected by centrifugation (800 × g, 10 min, 4*°*C) and used for RNA extraction according to the manufacturer’s protocol of RNeasy Mini Kit (Qiagen, Germany). RNA purity was assessed by calculation of the ratio between absorbance at 260 and 280 nm (A260/A280) using a NanoDrop spectrophotometer (ND-1000, Thermo Scientific, USA). The isolated RNAs were stored at -80 °C and then used for cDNA synthesis. Purified RNA was reverse transcribed to cDNA by the PrimeScript™ RT Reagent Kit (Takara, Japan) according to manufacturer’s protocol. All cDNAs were kept at -20 °C until further analysis.

### Quantitative real-time PCR (RT-qPCR)

The mRNA expression levels of interleukin-1β (IL-1β), IL-8, tumor necrosis factor α (TNF-α), transforming growth factor-beta (TGF-β), inducible nitric oxide synthase (iNOS), B-cell lymphoma 2 (Bcl-2), BCL-2-associated X protein (Bax), caspase-9, and caspase-3 genes in treated Caco-2 cells for 4, 8 and 24 h time periods were determined using RT-qPCR assays as previously described [35]. Gene expression was evaluated by the Rotor-Gene® Q (Qiagen, Germany) real-time PCR system using BioFACT™ 2X Real-Time PCR SYBR Green Master Mix (BIOFACT, South Korea). Oligonucleotide primers specific to each gene and their amplification conditions are presented in Supplementary Table 1. β-actin housekeeping gene served as the endogenous control. To confirm amplification specificity, a melting analysis and subsequent agarose gel electrophoresis were performed after each run. The relative gene expression data to β-actin were calculated according to the 2^− ΔΔCt^ method and presented as fold change to the control groups. All reactions were assessed in triplicate.

### Statistical analysis

GraphPad Prism 8.0 (GraphPad Software, CA, USA) was used for statistical analysis. Results were calculated and analyzed for statistical significance using Unpaired student’s t test and one-way analysis of variance (ANOVA). The data were presented as mean ± standard deviation (SD) for three independent experiments. Differences were considered statistically significant when *P* < 0.05; *P < 0.05, **P < 0.01, ***P < 0.001 and ****P < 0.0001.

## Results

### Phytochemical analysis of *A. millefolium* extracts

In this study, the phenolic and volatile compounds of *A. millefolium* were investigated by HPLC and GC, respectively. Chromatograms obtained from the analysis of DEC and ETOH extracts of *A. millefolium* are shown in Supplementary Figs. 1, 2, and 3. Phytochemical analysis of both extracts showed that ETOH extract contained more diverse phenolic compounds compared to DEC extract. Among eight phenolic compounds analyzed, the main compound identified in both extracts was chlorogenic acid (161.1 and 296.7 µg/mL in DEC and ETOH extracts, respectively). Interestingly, apigenin (7.0 µg/mL), rutin (55.4 µg/mL), and caffeic acid (4.1 µg/mL) were other antioxidants identified in ETOH extract, while in DEC extract, only apigenin (5.1 µg/mL) was detected (Table 1). The amount of other analyzed compounds, including gallic acid, catechin, quercetin, and rosmarinic acid, was not detectable in any of the extracts. Additionally, results of the quantitative measurement of three volatile compounds in DEC and ETOH extracts by GC analysis showed that ETOH extract contained two compounds, including limonene (4.9 µg/mL) and 1,8-cineol (16.3 µg/mL), while only limonene (5.7 µg/mL) was detected in DEC extract (Table 1).

**Table 1:** Phytochemical analysis of decocted (DEC) and ethanol (ETOH) extracts of *A. millefolium* detected by HPLC and GC

Analysis	Compounds	Rt (min)	Concentration (µg/mL)	
			DEC	ETOH
Phenolic compounds (HPLC)	Chlorogenic acid	8.08	161.1	296.7
	Caffeic acid	10.1	Nd	4.1
	Rutin	14.88	Nd	55.4
	Apigenin	21.85	5.1	7.0
Volatile compounds (GC)	Limonene	11.982	5.7	4.9
	1,8-cineol	12.191	Nd	16.3

^*^GC, gas chromatography; HPLC, high-performance liquid chromatography, Nd, not detected; Rt, retention time

### Antimicrobial susceptibility testing

The agar dilution method was performed to determine the antimicrobial activity of the extracts against *C. difficile* RT001, in which both extracts showed antimicrobial activity against this strain. The lowest MICs detected for DEC and ETOH extracts that inhibited the growth of RT001 were 75 µg/mL and 50 µg/mL, respectively. The extracts were further examined by the broth microdilution method to determine their MIC and inhibitory activities. Based on the results, the lowest MICs detected for DEC and ETOH extracts using broth microdilution method, which had complete inhibition on the growth of RT001, were 100 µg/mL and 75 µg/mL, respectively. The inhibitory activity of different concentrations of DEC and ETOH extracts against *C. difficile* RT001 are indicated in Table 2 and Supplementary Fig. 4.

**Table 2:** Percentage of inhibitory activity of decocted (DEC) and ethanol (ETOH) extracts of *A. millefolium* against *C. difficile* RT001 clinical strain

Concentration (µg/mL)	Extracts	
	ETOH (%)	DEC (%)
5	5.970	0
10	18.17	6.39
25	62.54	12.39
50	96.26	87.37
75	100	94.14
100	100	100
200	100	100

^*^RT, ribotype

### Effect of *C. difficile* RT001-derived Tox-S and *A. millefolium* extracts on cell viability in Caco-2 cells

**Fig. 1:**
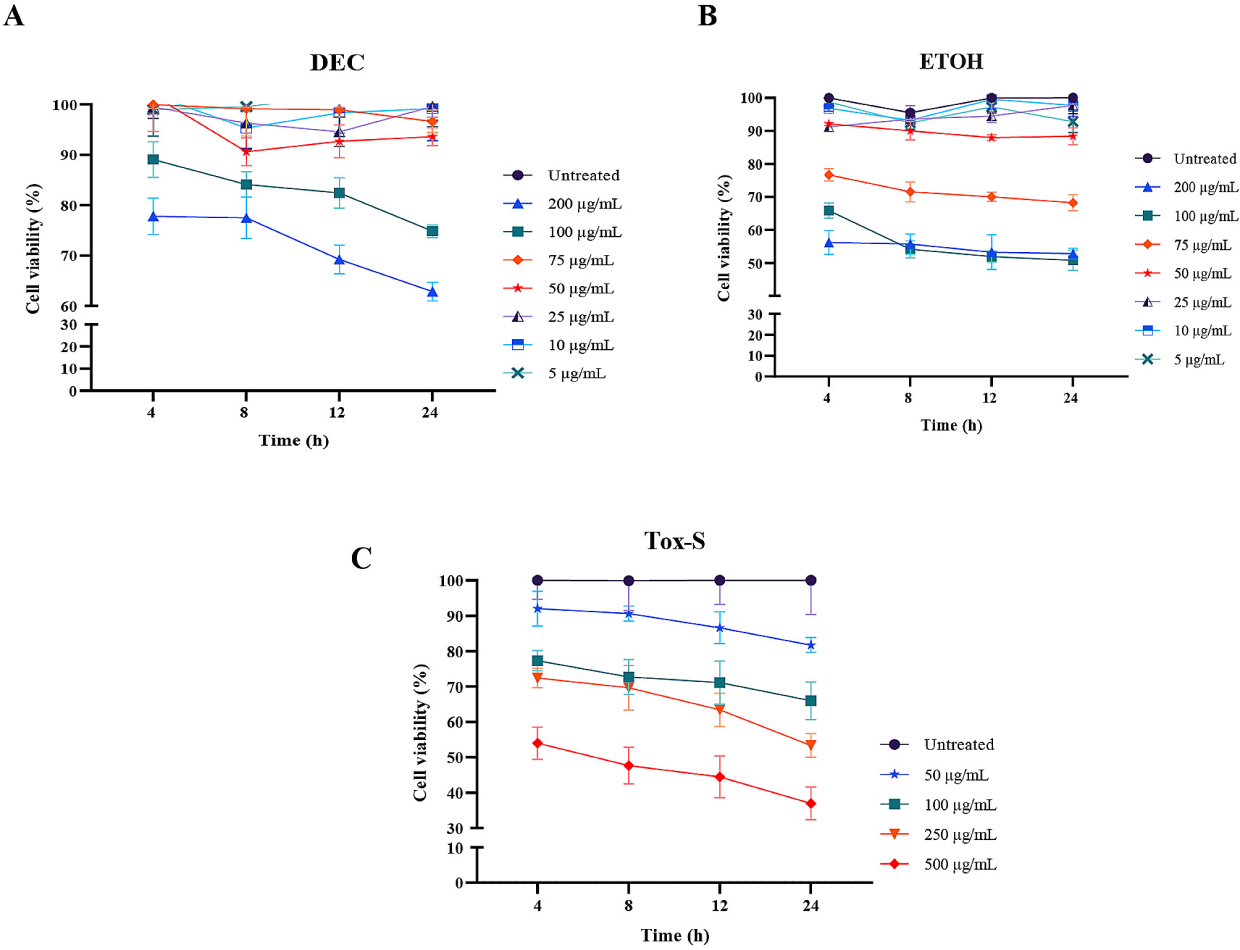
Cell viability of Caco-2 cells incubated with different concentrations (5, 10, 25, 50, 75, 100, and 200 µg/mL) of (**A**) decocted extract (DEC) and (**B**) ethanol extract (ETOH) of *A. millefolium*, (**C**) Tox-S (50, 100, 250, and 500 µg/mL) of *C. difficile* RT001 clinical strain. Data shown are means ± SD of three independent experiments

MTT assay was used to determine the cytotoxic effects of different concentrations of Tox-S, DEC, and ETOH extracts on Caco-2 cells for 4, 8, 12, and 24 h. As presented in Fig. 1A, viability of Caco-2 cells was significantly decreased after treatment with 50 to 500 µg/mL of Tox-S compared with untreated cells (*P* < 0.001). Moreover, Tox-S at concentration of 50 µg/mL led to 20% cell death, whereas a greater effect was observed for higher concentrations 100 and 500 µg/mL that resulted in nearly 30% and 70% of cell death, respectively.

In contrast, DEC extract at concentrations of 5 to 75 µg/mL did not significantly decrease the viability of Caco-2 cells after 4 to 24 h, whereas with an increase in the concentration of this extract the amount of cytotoxicity was augmented (Fig. 1B). For ETOH extract, no cytotoxic effect was seen up to concentration 50 µg/mL, while at higher concentrations the viability of cells decreased significantly (Fig. 1C).

### Cytotoxicity of *C. difficile* RT001-derived Tox-S and *A. millefolium* extracts on Vero cells

**Fig. 2:**
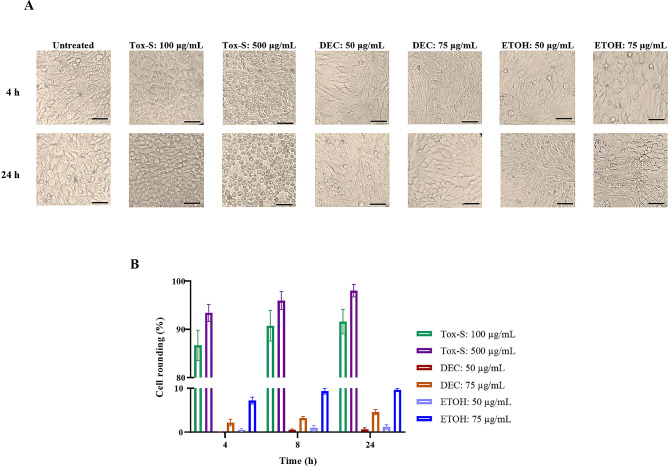
Cytopathic effect (CPE) of two different concentrations (100 and 500 µg/mL) of Tox-S from *C. difficile* RT001 clinical strain and two different concentrations (50 and 75 µg/mL) of decocted (DEC) and ethanol (ETOH) extracts on Vero cells. (**A**) Microscopic cell morphology of Vero cells after treatment with Tox-S, DEC and ETOH extracts for 4, 8, 24 h at 37 °C in comparison with untreated cells. Light microscopy (200X magnification), Scale bar = 100 μm. (**B**) Percentage of Vero cell rounding induced by Tox-S, DEC and ETOH extracts for 4, 8, 24 h at 37 °C in comparison with untreated cells. Data shown are means ± SD of three independent experiments

To evaluate the cytotoxicity of Tox-S and extracts on Vero cells, two concentrations of Tox-S and two concentrations of extracts were applied. After 4, 8, and 24 h, the percentage of cell rounding for each treatment was calculated by ImageJ software. The microscopic cell morphology of Vero cells showed that Tox-S of RT001 can induce CPE and cell rounding, in particular at 500 µg/mL (Fig. 2A). As shown in Fig. 2B, Tox-S at concentration of 100 µg/mL induced about 90% cell rounding after 24 h, while the highest CPE was observed at concentration 500 µg/mL of Tox-S, which led to 98% cell rounding compared to untreated cells. In contrast, no CPE was observed at concentration 50 µg/mL for both extracts after 4 and 8 h of treatment of Vero cells, while a small cytotoxicity was observed for both extracts after 24 h as compared with untreated cells. Interestingly, both DEC and ETOH extracts showed moderate CPE at concentration 75 µg/mL, which led to approximately 5% and 10% cell rounding after 24 h of treatment, respectively. Since a concentration of 50 µg/mL of both extracts had a lower CPE, further experiments were carried out with this concentration.

### Protective effect of *A. millefolium* extracts on Tox-S mediated cytotoxicity of *C. difficile* RT001 in Vero cells

**Fig. 3:**
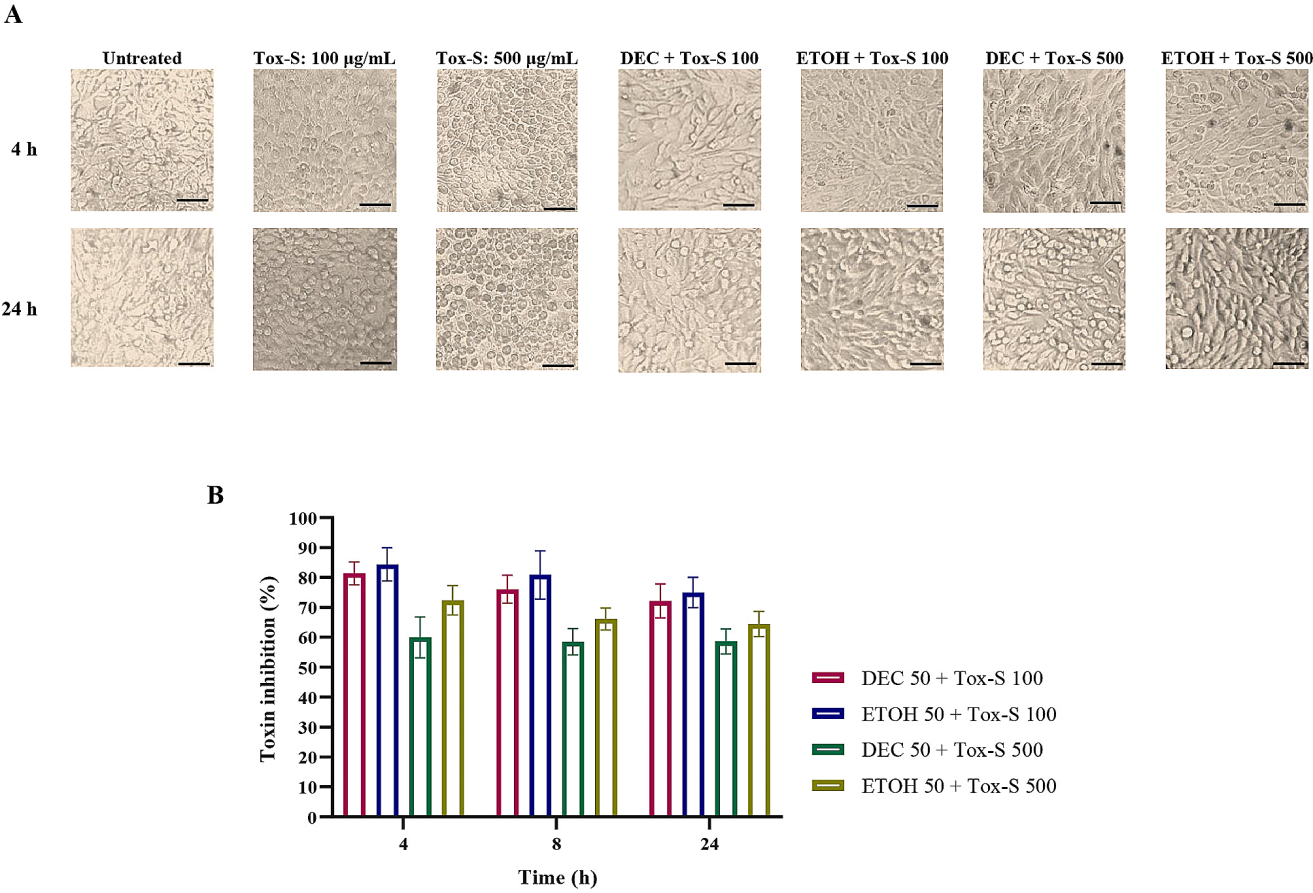
Inhibitory effects of decocted (DEC) and ethanol (ETOH) extracts (50 µg/mL) on cell rounding induced by two different concentrations (100 and 500 µg/mL) of Tox-S from *C. difficile* RT001 clinical strain on Vero cells. (**A**) Microscopic cell morphology of Vero cells co-treated with Tox-S and DEC or ETOH extracts for 4, 8, 24 h at 37 °C in comparison with untreated cells. Light microscopy (200X magnification), Scale bar = 100 μm. (**B**) Percentage of inhibition of Vero cell rounding induced by Tox-S and co-treated with DEC or ETOH extracts for 4, 8, 24 h at 37 °C in comparison with untreated cells. Number of round cells was normalized to the cells treated with Tox-S exhibiting 100% cytotoxicity. Data shown are means ± SD of three independent experiments

To evaluate the protective effect of plant extracts on Tox-S mediated cytotoxicity on Vero cells, two different concentrations of Tox-S (100 and 500 µg/mL resulting in approximately 90 and 98% cell rounding, respectively) were co-incubated with 50 µg/mL of DEC or ETOH extracts. The results of morphological examination showed that both extracts notably reduced the percentage of round cells induced by Tox-S compared to control cells (the percentage of round cells in the wells treated with Tox-S) (Fig. 3A). In more details, simultaneous treatment of cells with both concentrations of Tox-S and either DEC or ETOH extracts inhibited the cytotoxic effect of Tox-S by more than 50% (Fig. 3B). There was no significant difference between the inhibitory effect of DEC and ETOH extracts on cell rounding induced by different concentrations of Tox-S. However, Tox-S at concentration 100 µg/mL was applied for further gene expression experiments due to having less destructive effects on both cell lines used in this study.

### Effect of *C. difficile* RT001-derived Tox-S and *A. millefolium* extracts on gene expression of inflammatory parameters in Caco-2 cells

**Fig. 4:**
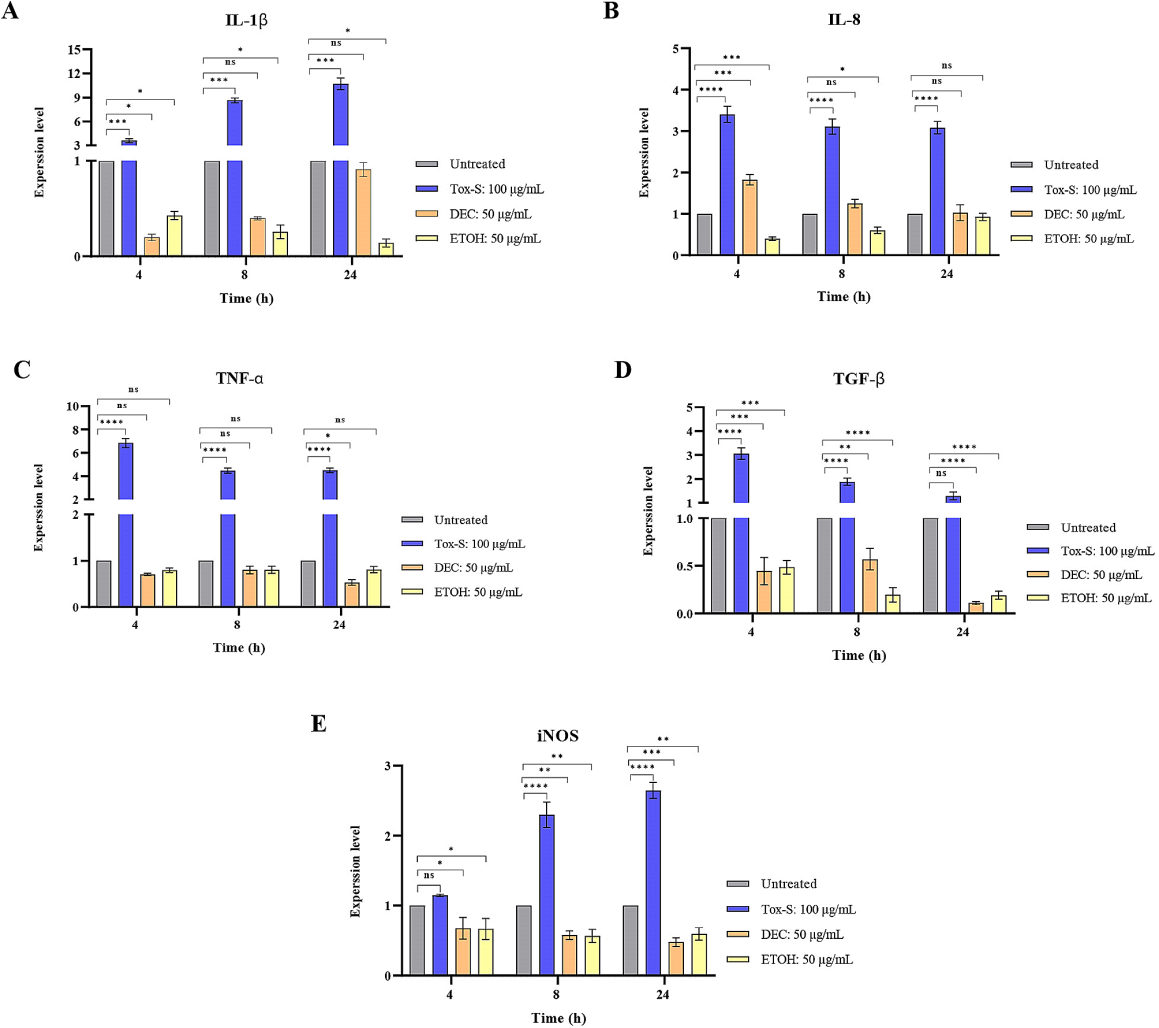
Relative expression of IL-1β (**A**), IL-8 (**B**), TNF-α (**C**), TGF-β (**D**), and iNOS (**E**) genes in Caco-2 cells upon treatment with Tox-S (100 µg/mL) from *C. difficile* RT001 clinical strain, decocted (DEC), and ethanol (ETOH) extracts (50 µg/mL) measured by using quantitative real-time PCR assay. Gene expression data was normalized to β-actin as the reference gene. Data shown are means ± SD of three independent experiments. A *P* value of < 0.05 was considered as significant (*P < 0.05; **P < 0.01; ***P < 0.001) by unpaired student’s t test and one-way ANOVA statistical analysis

The RT-qPCR assay was used to examine the effects of Tox-S and extracts on expression level of inflammation-associated genes in Caco-2 cells. As presented in Fig. 4, the expression level of IL-1β, IL-8, TNF-α, TGF-β, and iNOS was significantly increased upon treatment of Caco-2 cells with Tox-S compered to untreated cells. Inversely, treatment of Caco-2 cells with both extracts downregulated the expression level of over-mentioned inflammation-related genes, which showed statistically significant differences in some treatment groups. Although ETOH extract showed a stronger effect than DEC extract on downregulating expression level of indicated genes, this effect did not follow a time-dependent manner.

### Effect of *C. difficile* RT001-derived Tox-S and *A. millefolium* extracts on gene expression of apoptosis related genes in Caco-2 cells

**Fig. 5:**
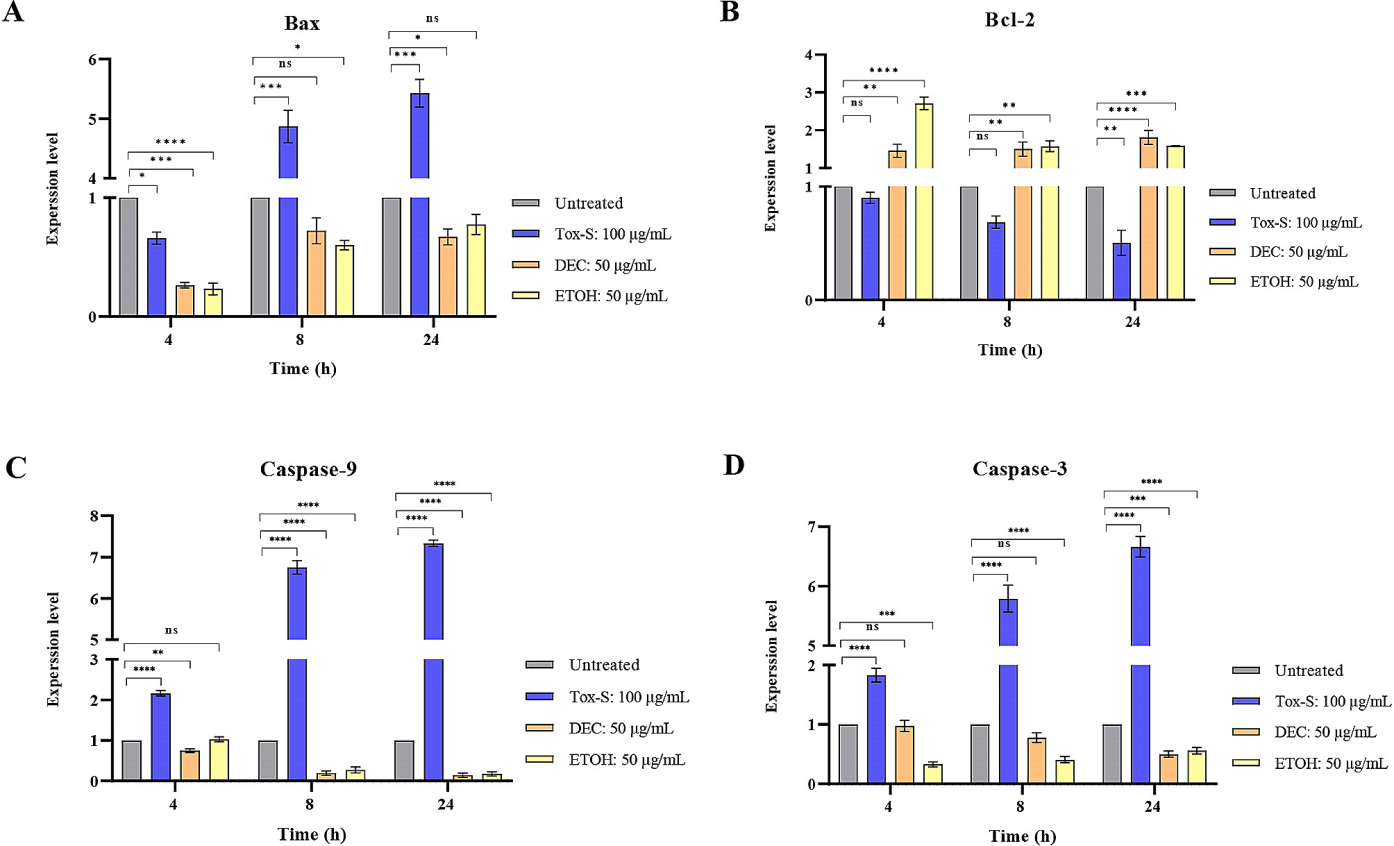
Relative expression of Bax (**A**), Bcl-2 (**B**), Caspase-9 (**C**), and Caspase-3 (**D**) genes in Caco-2 cells upon treatment with Tox-S (100 µg/mL) from *C. difficile* RT001 clinical strain, decocted (DEC), and ethanol (ETOH) extracts (50 µg/mL) measured by using quantitative real-time PCR assay. Gene expression data was normalized to β-actin as the reference gene. Data shown are means ± SD of three independent experiments. A *P* value of < 0.05 was considered as significant (*P < 0.05; **P < 0.01; ***P < 0.001) by unpaired student’s t test and one-way ANOVA statistical analysis

To determine the effect of Tox-S and extracts on apoptosis induction, the gene expression level of Bax, caspase-9, caspase-3, and Bcl-2 was assessed in treated Caco-2 cells during the indicated time points. As shown in Fig. 5, the gene expression level of Bax, caspase-9, and caspase-3 was significantly induced by Tox-S after 8 and 24 h, while it was downregulated in Caco-2 cells treated with both DEC and ETOH extracts. In contrast, the gene expression level of Bcl-2 was significantly downregulated by Tox-S in treated Caco-2 cells compared to untreated cells after 24 h, whereas it was significantly upregulated upon treatment with both extracts after the indicated time periods.

### Modulatory effect of *A. millefolium* extracts on expression level of inflammatory related genes in Tox-S treated Caco-2 cells

**Fig. 6:**
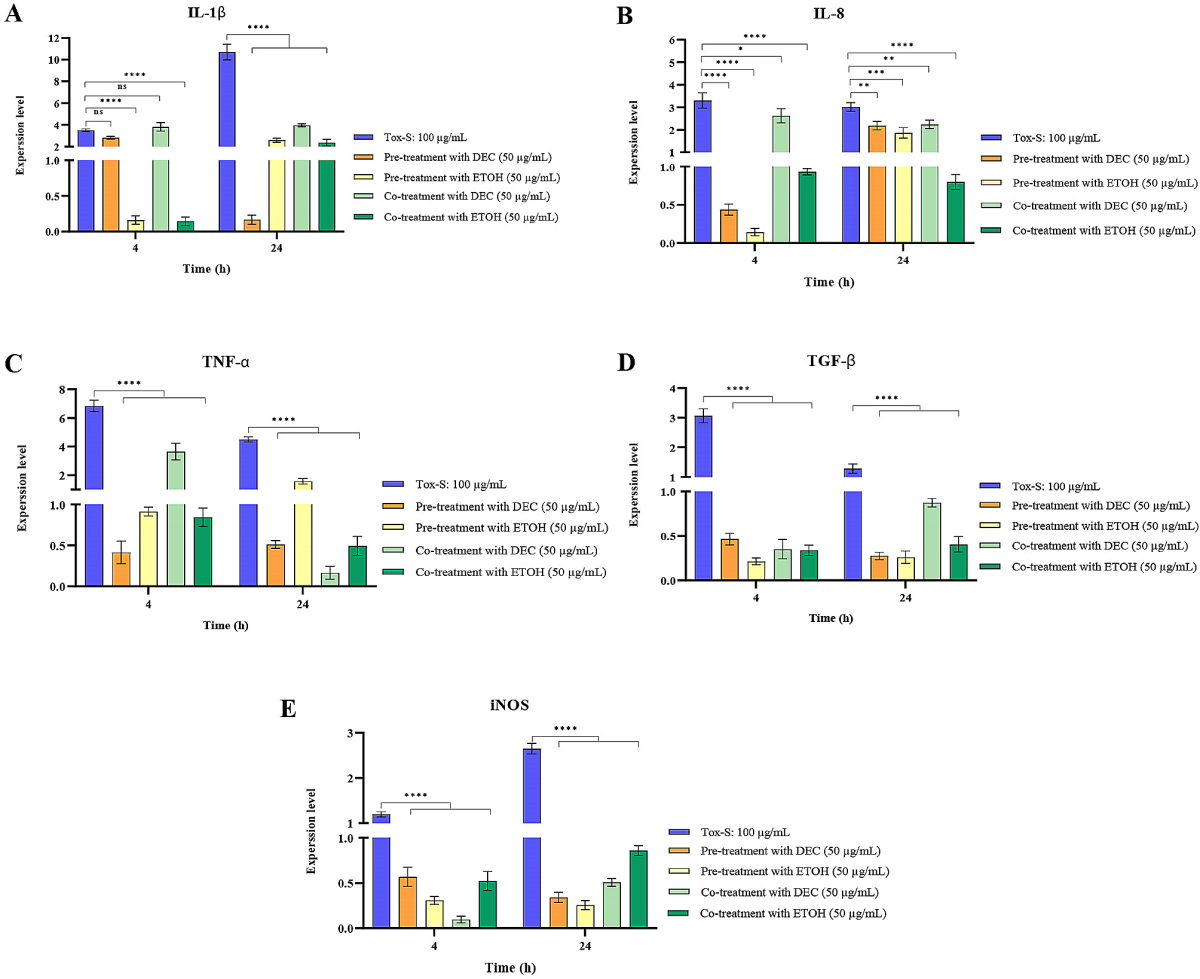
Relative expression of IL-1β (**A**), IL-8 (**B**), TNF-α (**C**), TGF-β (**D**), and iNOS (**E**) genes in Caco-2 cells upon pre-treatment with decocted (DEC) or ethanol (ETOH) extracts (50 µg/mL), followed by treatment with Tox-S (100 µg/mL) from *C. difficile* RT001 clinical strain; and also co-treatment with DEC and Tox-S, or ETOH and Tox-S measured by using quantitative real-time PCR assay. Gene expression data was normalized to β-actin as the reference gene. Data shown are means ± SD of three independent experiments. A *P* value of < 0.05 was considered as significant (*P < 0.05; **P < 0.01; ***P < 0.001) by unpaired student’s t test and one-way ANOVA statistical analysis

To evaluate the anti-inflammatory effects of both extracts on Tox-S treated Caco-2 cells, the expression level of inflammation-associated genes was determined in pre-treated and co-treated cells with each extract. As shown in Fig. 6, pre-treatment of Caco-2 cells with DEC or ETOH extracts significantly decreased the expression level of IL-1β, IL-8, TNF-α, TGF-β, and iNOS genes induced by Tox-S almost in a time-independent manner. Similarly, both extracts markedly decreased the gene expression level of indicated inflammation-related markers upon co-treatment of Caco-2 cells with Tox-S and extracts in a time-independent manner.

### Modulatory effect of *A. millefolium* extracts on expression level of apoptosis related genes in Tox-S treated Caco-2 cells

**Fig. 7:**
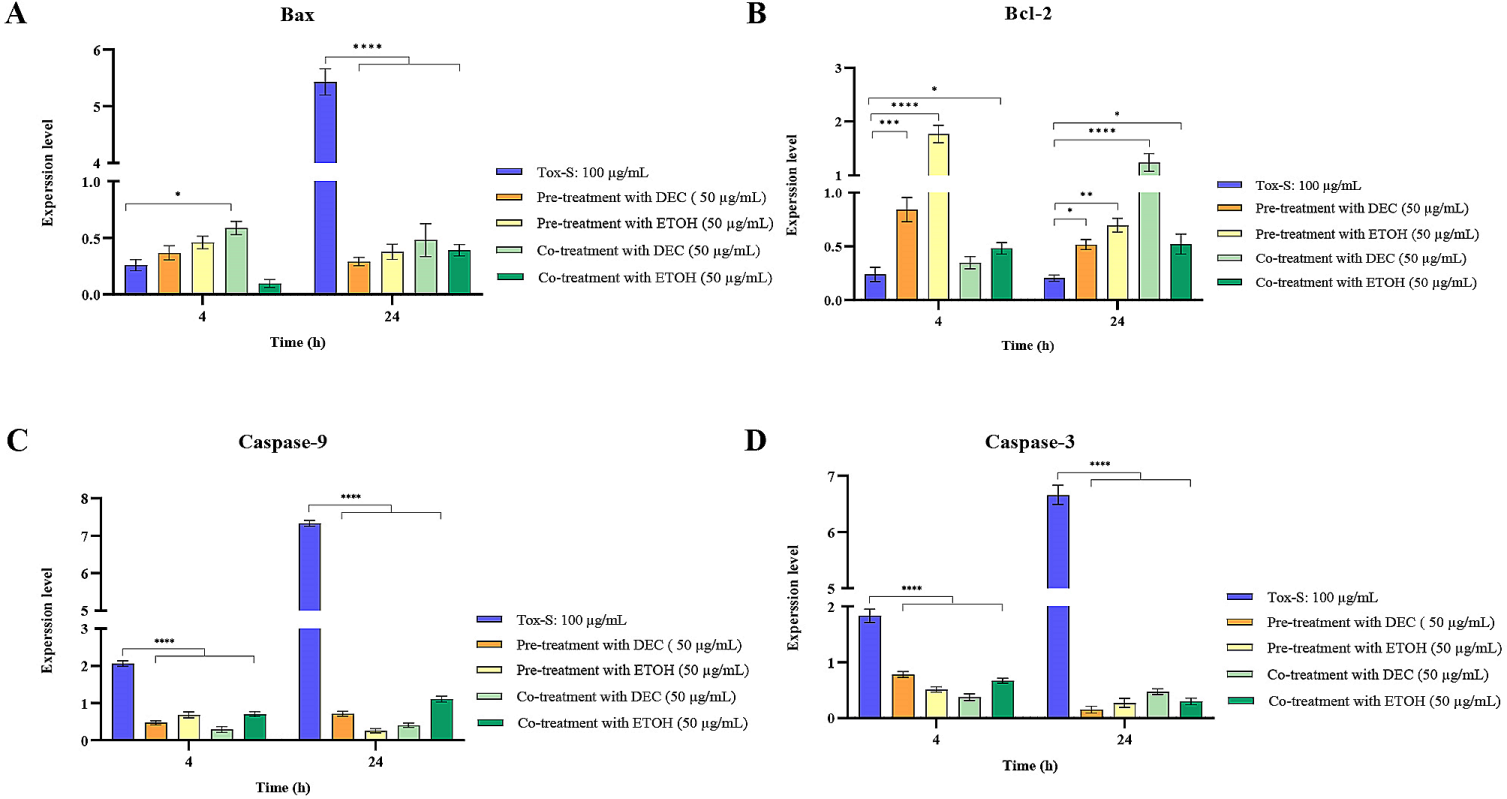
Relative expression of Bax (**A**), Bcl-2 (**B**), Caspase-9 (**C**), and Caspase-3 (**D**) genes in Caco-2 cells upon pre-treatment with decocted (DEC) or ethanol (ETOH) extracts (50 µg/mL), followed by treatment with Tox-S (100 µg/mL) from *C. difficile* RT001 clinical strain; and also co-treatment with DEC and Tox-S, or ETOH and Tox-S measured by using quantitative real-time PCR assay. Gene expression data was normalized to β-actin as the reference gene. Data shown are means ± SD of three independent experiments. A *P* value of < 0.05 was considered as significant (*P < 0.05; **P < 0.01; ***P < 0.001) by unpaired student’s t test and one-way ANOVA statistical analysis

To determine the anti-apoptosis effects of both extracts on Tox-S treated Caco-2 cells, the expression level of Bax, caspase-9, caspase − 3 and Bcl-2 genes was assessed in pre-treated and co-treated cells with each extract. As shown in Fig. 7, pre-treatment and co-treatment of Caco-2 cells with DEC or ETOH extracts significantly decreased the gene expression level of Bax, caspase-9, and caspase− 3 induced by Tox-S during the indicated time points. In contrast, extracts in both types of treatment, pre-treatment and co-treatment, significantly upregulated the gene expression level of Bcl-2 in Caco-2 cells stimulated with Tox-S after 4 and 24 h.

## Discussion

Globally, CDI is reported as a considerable threat to both public health and healthcare setting leading to substantial economic and medical costs [4]. Although the preferred approved management of patients with CDI is antibiotic administration, the use of antibiotics can result in profound alterations to the intestinal microbiota structure and disrupt colonization resistance predisposing the host to rCDI [38]. The toxigenic *C. difficile* RT001 (A^+^B^+^) has been identified as the most common frequent RT types detected in Iran [34, 39]. The majority of the strains belonging to RT001 are multidrug resistant [39], thus developing novel alternative approaches to antibiotic treatment are crucial for prevention and control of RT001 in this area. Additionally, the development of novel alternative therapeutic components that can simultaneously maintain the gut homeostasis and tackle pathogens and neutralize their toxins is of great importance for improving clinical outcomes and reducing the recurrence rates of CDI.

So far, several studies have been conducted to examine the inhibitory effects of *A. millefolium* extracts on different bacterial species [28, 29, 40]. Different techniques can be used to extract bioactive compounds, among which maceration in ethanol solvent is a simple and low-cost method to extract the major components of plants [41]. Additionally, the decoction is a traditional and easily available method used for the extraction of medicinal plants [27]. The potential of protective effects and beneficial features of *A. millefolium* led us to the hypothesis that the use of different extracts of this plant, i.e., DEC and ETOH extracts, could modulate *C*. *difficile* Tox-S mediated cytotoxicity in vitro. Further, the anti-inflammatory and anti-apoptosis activities of *A. millefolium* DEC and ETOH extracts were investigated using a human colon cancer cell line treated with *C*. *difficile* Tox-S. The results of both agar dilution and broth microdilution assays revealed potent inhibitory activities of *A. millefolium* extracts on the growth of *C. difficile* RT001. These results are relatively in agreement with the study performed by Karaalp et al. where they showed that *A. millefolium* extracts could exert a minimal anti-microbial activity of ≥ 100 µg/mL against different bacteria, including *Bacillus cereus*, *Salmonella typhimurium*, and *Salmonella* Agona [42]. Interestingly, they also reported that essential oil of *A. millefolium* showed stronger anti-microbial activity for Gram-positive bacteria than Gram-negative bacteria. In another study by Candan et al. [28], the antibacterial properties of *A. millefolium* oil collected from Turkey were also examined against *B. cereus* and *S. aureus*, showing MIC value of 72 mg/mL, which was almost similar to the MIC values determined in our study. It has been reported that the antibacterial activity of *A. millefolium* is contributed to its components including chlorogenic acid, caffeic acid, 1,8-cineole, cis-sabinene hydrate, α-terpinol, α-cadinol, terpinen-4-ol, p-cymene, and camphor [43]. Apparently, the hydrophobicity property of *Achillea* components targets the cell membrane and increases the permeability of bacterial cells [44, 45]. The phytochemical analysis of extracts revealed that chlorogenic acid, apigenin, and limonene were detected in both extracts, whereas rutin, caffeic acid, and 1,8-cineol were only detected in ETOH extract. Interestingly, the extractive yield was greater in ETOH extract than DEC extract, which is also supported by other researchers [24, 27, 43]. The differences detected on the overall bioactive components of two extracts partially could be contributed to use of different extraction methods [27, 41, 46]. Given the hydrophobicity property of these chemicals, the primary target of these components is the cell membrane. These components can penetrate microbial cells and cause loss of integrity and increased permeability, resulting in bacterial cellular leakage and death [44]. Earlier studies also reported that caffeic and chlorogenic acids could exert high antimicrobial activities against several bacterial species [47–49]. Interestingly, it has been documented that the constituents of essential oils can apply synergistic effects on different pathogens [50]. Based on our results, it can be suggested that chlorogenic acid, caffeic acid, 1,8-cineol, and limonene may be involved in the antimicrobial activity of the extracts examined in the present study. However, further research using purified components of *A. millefolium* is required to discover their precise inhibitory mode of action against *C. difficile* cells.

The cell viability results demonstrated that different concentrations of both DEC and ETOH extracts had no significant adverse effect on viability of Caco-2 cells. However, higher concentration of DEC extract showed a lower CPE than ETOH. This could be due to higher dissolution of some components in decoction method compared to maceration process, relating to high temperature used in the decoction process. The high temperature leads to the transformation of diester-diterpenoid alkaloids (DDAs) to monoester-diterpenoid alkaloids (MDAs) having less toxic effects [41]. Our results supported previous reports about the low in vitro [51–53] and in vivo [53] cytotoxicity of different extracts of *Achillea* species. Additionally, other studies have demonstrated that the presence of phenolic components, especially chlorogenic acid, can induce apoptotic activity in different cell lines and act as an anti-cancer agent [54, 55]. This can explain the cytotoxic activities reported for higher concentrations of the extracts.

It has been well established that both TcdA and TcdB from *C. difficile* strains can affect IECs through inactivation of Rho/Ras proteins and induce cell apoptosis both in vitro and in vivo [36, 56, 57]. Our results indicated that treatment of the Vero cells with concentration of 100 µg/mL of Tox-S can stimulate 90% cell rounding. Interestingly, the co-treatment of *Achillea* extracts could strongly reduce the cell rounding of Vero cells stimulated by Tox-S. Moreover, no significant difference was observed for the ability of DEC or ETOH extracts in reducing cytotoxicity caused by Tox-S. It has been proposed that some plant extracts may act as a physical barrier and/or directly interact with toxins, thus reducing the exposure of cells to toxins and prevent their internalization [58, 59].

As shown in previous studies, TcdB significantly activates MAPKs, NF-κB and subsequently induces the production of IL-1β and TNF-α [8, 60]. Additionally, TGF-β is a multifunctional cytokine that regulates various cellular processes like cell growth, adhesion, differentiation, apoptosis, and immunosuppression [61]. Previous data show that TcdA is able to increase the expression of TGF-β1 and its receptor, TβRII, both in IEC cells and in mouse ileal tissue [62]. Based on our results, exposure of Caco-2 cells with Tox-S of *C. difficile* RT001 could upregulate the gene expression level of inflammatory cytokines. According to several previous studies, *A. millefolium* extracts exerted various effects on the transcriptional response of cells stimulated by LPS [18, 63]. Chou et al. demonstrated that *Achillea* extract can modulate inflammatory responses induced by LPS in RAW 264.7 murine macrophages through inhibition of oxidative stress, and by down-regulation of iNOS, COX-2, TNF-α and IL-6 expression [64]. In this work, both extracts decreased the gene expression level of a select number of genes engaged in inflammation and apoptosis pathways. It has been proven that the expression of inflammatory cytokines is extensively regulated by the NF-κB signaling pathway [17]. Accordingly, we assume that *A. millefolium* extracts may probably modulate the NF-κB activation and its downstream signaling mediators. Furthermore, the anti-inflammatory properties of *A. millefolium* could be attributed to secondary metabolites, such as flavonoids, alkaloids, isoprenoids, and phenolics, which can be present in its extracts at different concentrations [26].

Nitric oxide (NO) is one of the toxic chemical species released by the host epithelial cells during CDI, and acts as an early signal to activate inflammatory response and production of cytokines [65]. The overproduction of NO is related to elevated activation of iNOS that results in inflammation and intestinal injury [66]. It has been shown that TcdB of *C. difficile* can induce the expression of iNOS in vascular smooth muscle cells (VSMCs) [65]. Conversely, it has been reported that *Achillea* species could decrease NO levels [64], which is corroborated by our results. According to literature, many plant extracts can suppress NF-κB activation [17, 67, 68], which can be a probable underlying mechanism to explain the downregulatory impact of the extracts on iNOS expression in the present work as well.

In addition to inflammation, increased activity of iNOS can affect the apoptosis pathway and act as a mediator of apoptosis in cells [69]. The key regulating members of the Bcl-2 family, Bax and Bcl-2, are actively involved in promoting or inhibiting apoptotic pathways triggered by mitochondrial dysfunction [70]. The overexpression of Bax, known as a pro-apoptotic agent, occurs in response to different cellular stresses and activates a cascade of reactions by releasing cytochrome C from the mitochondria, leading to the activation of caspase-9 and caspase− 3, ultimately resulting in apoptosis [71]. In contrast, Bcl-2 acts as a barrier to apoptosis and restricts most types of apoptotic cell death by suppressing the Bax activity [70]. Interestingly, caspase-3 can cleave Bcl-2, which activates a positive feedback loop for reinforcing the apoptotic effect [72]. It has been well established that both TcdA and TcdB from *C. difficile* strains can induce cell apoptosis in vitro and in vivo [7, 57]. Similarly, our results revealed that Tox-S extracted from *C. difficile* RT001 can modulate the gene expression of Bax, caspase-9, caspase-3 and Bcl-2, leading to induction of apoptosis in Caco-2 cells in a time-dependent manner. In contrast, exposure of Tox-S treated Caco-2 cells with both extracts caused a significant decrease on the mRNA expression level of Bax, caspase-9, caspase-3 and a significant increase the expression level of Bcl-2. These results indicate that *A. millefolium* extracts can possibly inhibit apoptosis through inactivation of the Bax-caspase-9-caspase-3 axis. There a limited number of studies demonstrating the anti-apoptosis activity of plant products [73, 74]. In addition, several studies have shown that *Achillea* can trigger apoptosis in various cancer cell types, indicating that *Achillea* profits in a dual manner in proceeding cell apoptosis, albeit its precise molecular mechanism remains to be elucidated [31, 32]. It has been proposed that higher concentration of *Achillea* extracts can induce apoptotic cell death in different cancer cell lines through upregulating the expression level of Bax and caspase-3 genes and downregulating Bcl-2 expression [75, 76]. Inversely, Okkay et al. reported that *A. millefolium* extracts can suppress apoptosis through reducing the expression of caspase-3 in Wistar rats [77]. This finding is in line with our results, suggesting an anti-apoptotic effect of *Achillea* extracts in Tox-S treated cells. Additionally, several pharmacological experiments have proved the potential of *A. millefolium* extracts with anti-inflammatory, antiulcer, and anticancer activities with varying doses (400 to 1600 mg/kg/day) [78, 79]. However, further research using in vivo models and clinical experiments is warranted to discover pharmacokinetic profile of *A. millefolium* extracts in the intestine, and to clearly define safety, efficacy, and inhibitory effects of these extracts against *C. difficile*.

## Conclusion

**Fig. 8:**
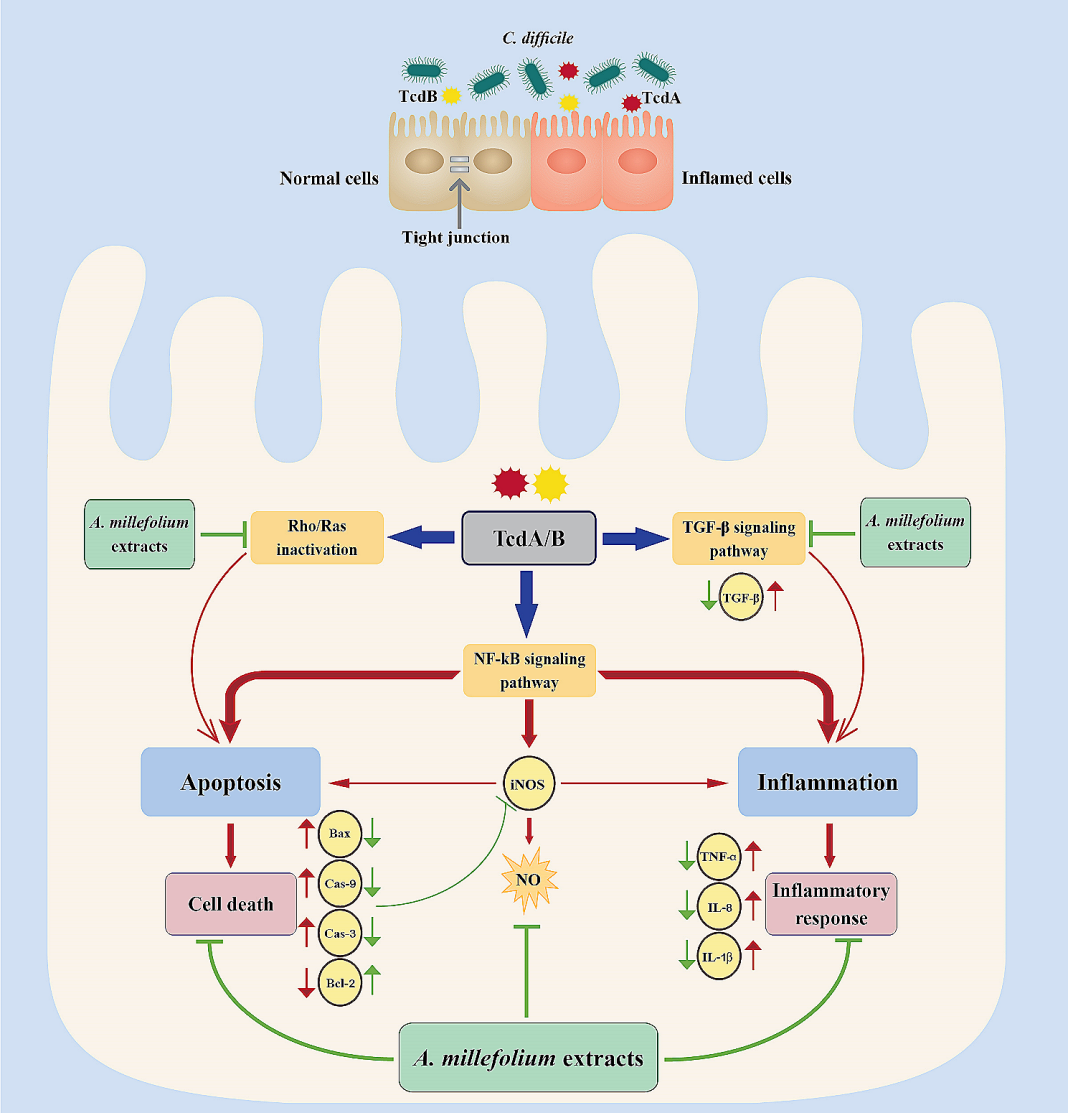
A schematic diagram that demonstrates the potential biological impact of *A. millefolium* extracts on *C. difficile* toxin-mediated inflammation. Exposure of intestinal epithelial cells (IECs) to *A. millefolium* extracts can lead to inhibition of NF-κB and TGF-β signaling pathways, which may decrease the expression of inflammatory cytokines like TNF-α, IL-8, and IL-1β. In addition, inhibition of iNOS activity by extracts can be one of the potential modulatory mechanisms affecting the suppression of apoptosis and inflammatory pathways mediated by *C. difficile* major toxin*s*. On the other hand, *A. millefolium* extracts can inhibit inactivation of Rho/Ras proteins induced by toxin*s*, resulting in apoptosis suppression. Moreover, they may inhibit apoptosis via downregulation of Bax and upregulation of Bcl-2, and then inactivate caspase cascades. Notes: red arrows indicate enhancing actions induced by toxin*s*, whereas green arrows indicate inhibitory actions induced by *A. millefolium* extracts. Bax: Bcl-2-associated X protein; Bcl-2: B-cell lymphoma 2; Cas-9: Caspase-9; Cas-3: Caspase-3; IECs: intestinal epithelial cells; IL-8: interleukin-8, IL-1β: interleukin-1β; iNOS: inducible nitric oxide synthase; NF-κB: nuclear factor kappa B; TGF-β: transforming growth factor-β; TNF-α: tumor necrosis factor α

In conclusion, the results of the present study for the first time showed that both DEC and ETOH extracts obtained from *A. millefolium* can inhibit the growth of *C. difficile* RT001. Although both extracts exhibited low cytopathic effects on Caco-2 and Vero cells, DEC extract exerted safer biological activity compared to ETOH extract due to its lower cytotoxicity. This finding indicates that the type of extraction method used can affect the cytotoxicity of herbal extracts. In addition, our findings demonstrated that both extracts can exert anti-inflammatory and anti-apoptosis activity in Caco-2 cells stimulated by Tox-S of *C. difficile* RT001. We propose that these modulatory effects are possibly elicited through suppressing the activation of key pathways involved in *C. difficile* toxin-mediated inflammation, including NF-κB and TGF-β signaling pathways, and also inhibiting the Rho/Ras inactivation pathway engaged in apoptosis regulation (Fig. 8). One of the major limitations of the present work refers to restriction in the composition analysis of the extracts, where only 11 plant compounds were determined. Furthermore, this work provides preliminary evidence for antimicrobial, anti-inflammatory, and anti-apoptosis activities of *A. millefolium* extracts at in vitro level. Further research using animal models is required to precisely evaluate the toxicity, efficacy and inhibitory effects of these extracts on *C. difficile* growth and pathogenesis. Taken together, the advocacy of *A. millefolium* extracts to be applied as potential supplementary medicine to current therapies for rCDI needs to be explored in clinical.

### Electronic supplementary material

Below is the link to the electronic supplementary material.

Supplementary Material 1

## Data Availability

The data that support the findings of this study are available from the corresponding author upon reasonable request.

## Abbreviations

AAD: Antibiotic-associated diarrhea
ANOVA: One-way analysis of variance
Bcl-2: B-cell lymphoma 2
Bax: Bcl-2-associated X protein
BHI: Brain heart infusion broth
CCFA: Cycloserine-cefoxitin-fructose agar
CDI: *Clostridioides difficile* infection
CPE: Cytopathic effect
DMSO: Dimethyl sulfoxide
DDAs: Diester-diterpenoid alkaloids
DEC: Decoction
ELISA: Enzyme-linked immunosorbent assay
ETOH: Ethanol
FBS: Fetal bovine serum
FMT: Fecal microbiota transplantation
GC: Gas chromatography
H-DMEM: High-glucose Dulbecco’s modified Eagle’s medium
HPLC: High Performance Liquid Chromatography
IBD: Inflammatory bowel disease
IBRC: Iranian Biological Resource Center
IECs: Intestinal epithelial cells
IL-1β: Interleukin-1β
IL-8: Interleukin-8
iNOS: Inducible nitric oxide synthase
MDAs: Monoester-diterpenoid alkaloids
MIC: Minimum inhibitory concentration
MTT: 3-(4,5 dimethylthiazol-2-yl)-2,5-diphenyl tetrazolium bromide
NO: Nitric oxide
OD: Optical density
PBS: Phosphate-buffered saline
PDA: Photodiode array detector
PMC: Pseudomembranous colitis
RT-qPCR: Quantitative real-time PCR
rCDI: Recurrent *Clostridioides difficile* infection
rpm: Revolutions per minute
SD: Standard deviations
TcdA: Toxin A
TcdB: Toxin B
TGF-β: Transforming growth factor-β
TNF-α: Tumor necrosis factorα
Tox-S: Toxigenic cell-free supernatants
VSMCs: Vascular smooth muscle cells
