# Supplementary material for: The anti-inflammatory and anti-apoptotic effects of Achillea millefolium L. extracts on Clostridioides difficile ribotype 001 in human intestinal epithelial cells
Source: BMC Complement Med Ther. 2024 Jan 13;24:37. doi: 10.1186/s12906-024-04335-2 (PMC10790267; doi:10.1186/s12906-024-04335-2)
Supplement: Supplementary file 1 — Supplementary Material 1 [file 12906_2024_4335_MOESM1_ESM.pdf]

Supplementary materials

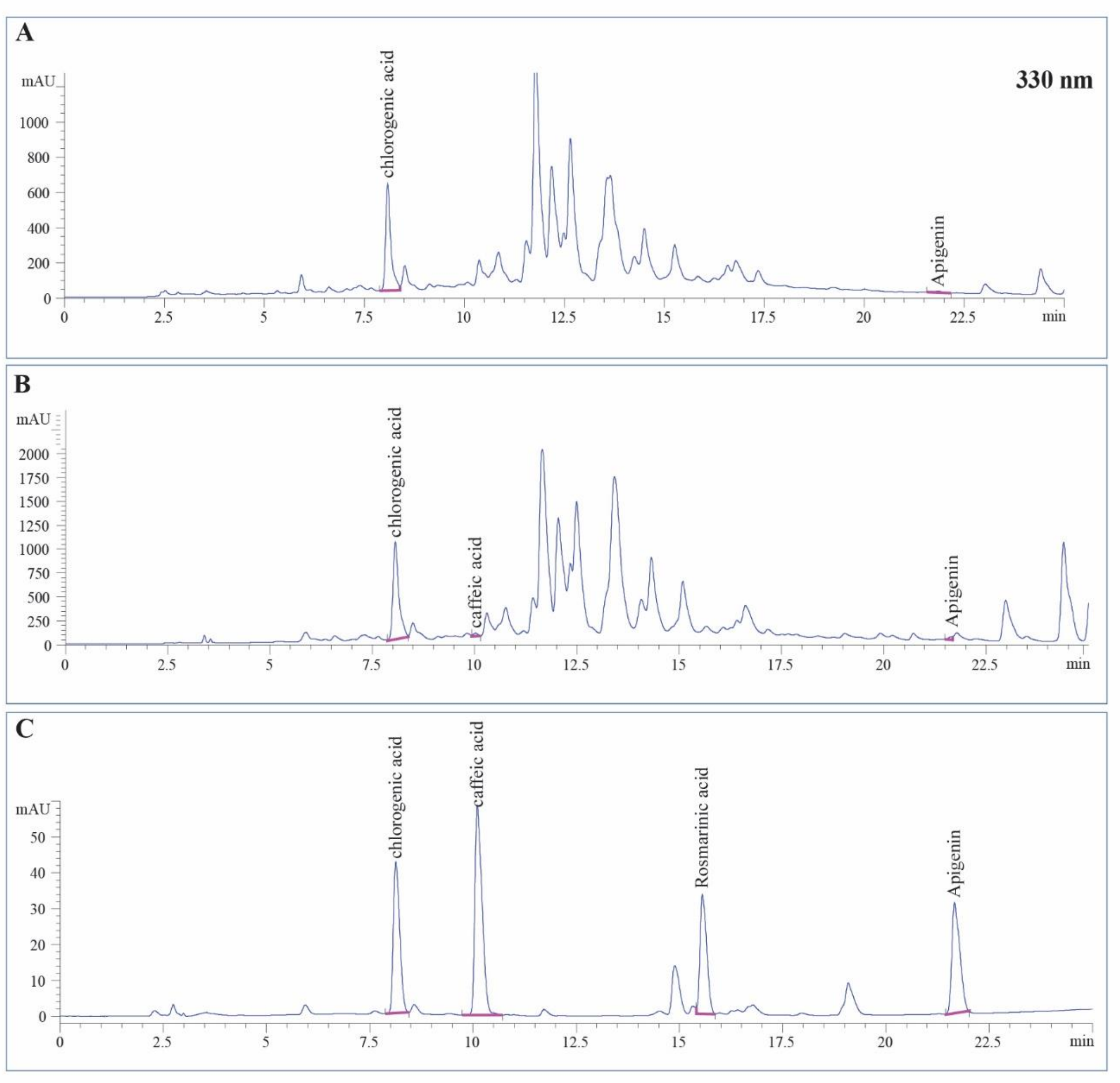

Supplementary Fig. 1. Typical HPLC chromatogram of *Achillea millefolium* extracts. Key components were identified based on the identical retention times as those of the standards for chlorogenic acid, caffeic acid, rosmarinic acid, and apigenin. The chromatograms of (**A**) decocted (DEC) extract, (**B**) ethanol (ETOH) extract, and (**C**) standards in 330 nm.

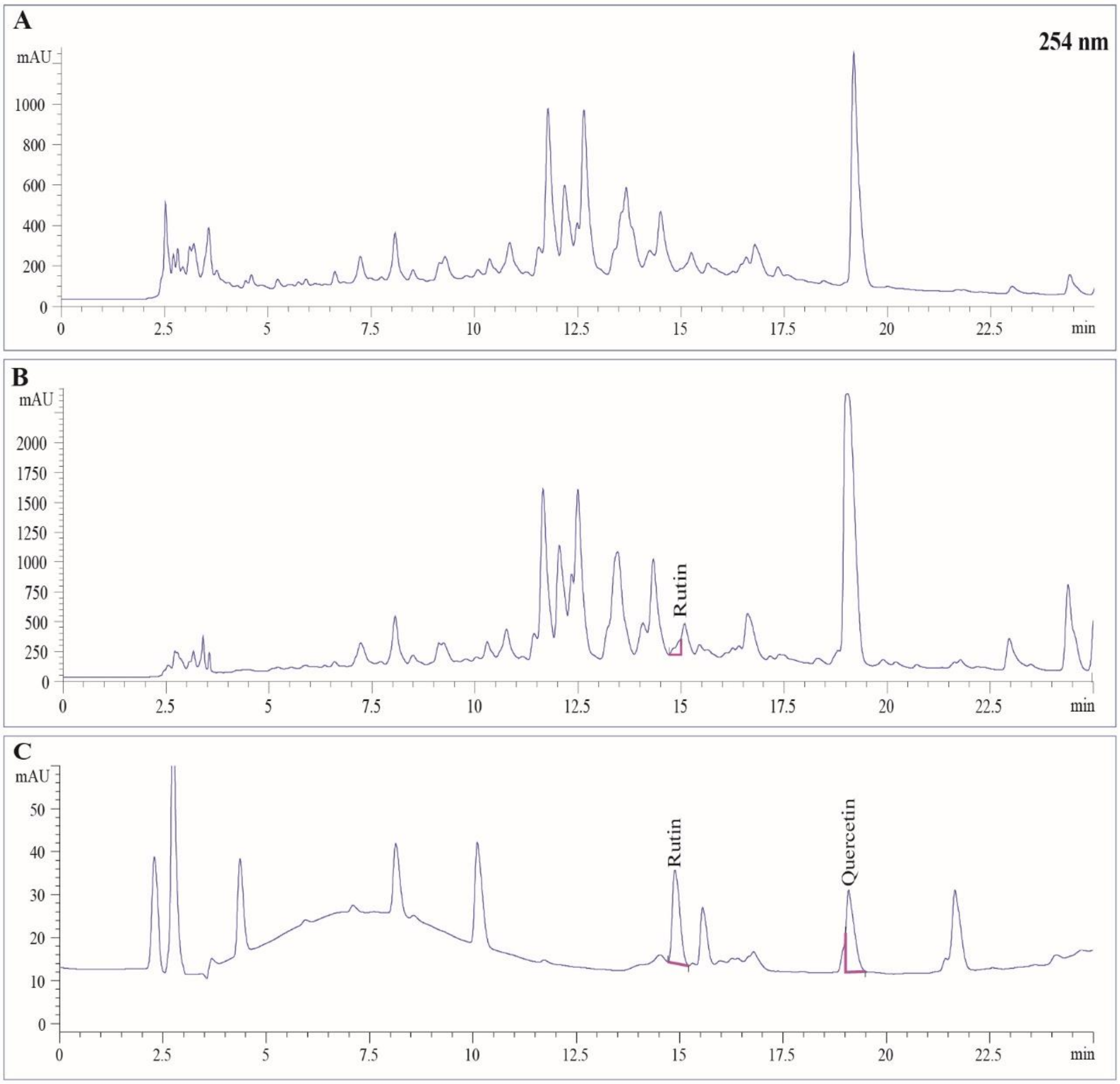

Supplementary Fig. 2. Typical HPLC chromatogram *of Achillea millefolium* extracts. Key components were identified based on the identical retention times as those of the standards for rutin and quercetin. The chromatograms of (**A**) decocted (DEC) extract, (**B**) ethanol (ETOH) extract, and (**C**) standards in 254 nm.

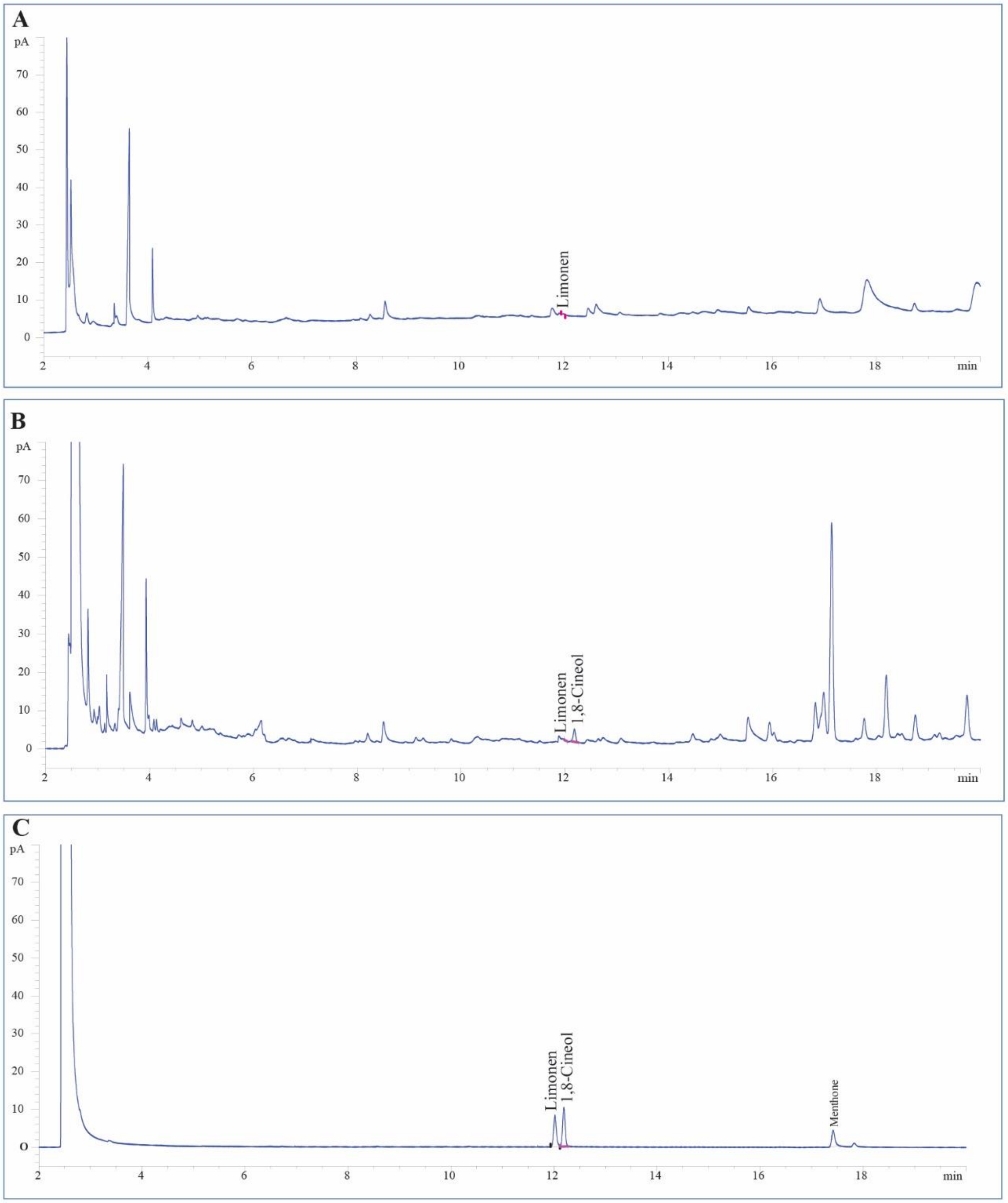

Supplementary Fig. 3. Typical GC chromatogram of *Achillea millefolium* extracts. Key components were identified based on the identical retention times as those of the standards for limonene, 1,8-cineol, and menthone. The chromatograms of (**A**) decocted (DEC) extract, (**B**) ethanol (ETOH) extract, and (**C**) standards.

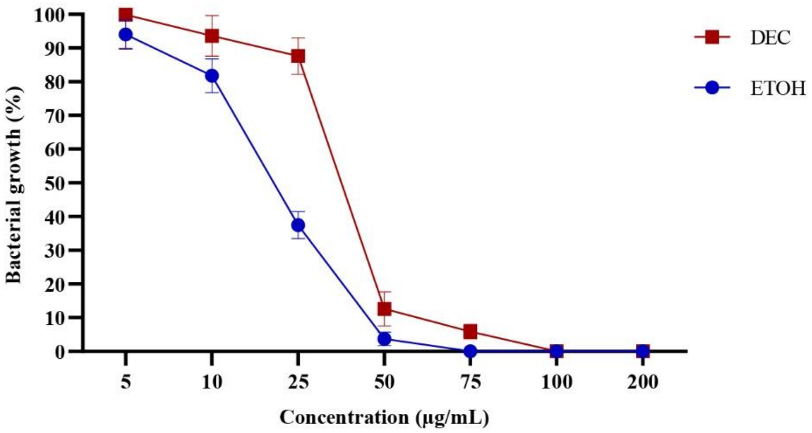

Supplementary Fig. 4. Inhibitory activity of different concentrations of decocted (DEC) and ethanol (ETOH) extracts of *Achillea millefolium* against *C. difficile* RT001 clinical strain.

Supplementary Table 1 Oligonucleotide sequences used in this study.

Target gene	Oligonucleotide sequence (5′-3′)	T_m ºC	Reference
IL-1β	F: AACAACTACTCAGAAACACAAG<br>R: CAGAACTCAGGAATGGA	59	[1]
IL-8	F: AGCACTCCTTGGCAAAACTG<br>R: CGGAAGGAACCATCTCACTG	60	[2]
TNF-α	F: AGCCCATGTTGTAGCAAACC<br>R: TGAGGTACAGGCCCTCTGAT	56	[3]
TGF-β	F: CAATTCCTGGCGATACCTCAG<br>R: GCACAACTCCGGTGACATCAA	56	[4]
iNOS	F: AGACTGGATTTGGCTGGTCCCTCC<br>R: AGAACTGAGGGTACATGCTGGAGCC	56	[5]
Bax	F: CCTGTGCACCAAGGTGCCGGAACT<br>R: CCACCCTGGTCTTGGATCCAGCCC	56	[6]
Bcl-2	F: GAGCTGGTGGTTGACTTTCTC<br>R: TCCATCTCCGATTCAGTCCCT	56	[6]
caspase-9	F: CATATGATCGAGGACATCCAG<br>R: TTAGTTCGCAGAAACGAAGC	57	[7]
caspase-3	F: ACATGGCGTGTCATAAAATACC<br>R: CACAAAGCGACTGGATGAAC	57	[7]
β-actin	F: ATGTGGCCGAGGACTTTGATT<br>R: AGTGGGGTGGCTTTTAGGATG	59	[8]

References

1. Raftar SKA, Ashrafian F, Abdollahiyan S, Yadegar A, Moradi HR. Masoumi M, Vaziri F, Moshiri A, Siadat SD, Zali MR. The anti-inflammatory effects of Akkermansia muciniphila and its derivates in HFD/CCL4-induced murine model of liver injury. Sci Rep. 2022; 12(1): 2453. https://doi.org/10.1038/s41598-022-06414-1

2. Zhao S, Guo J, Zhao Y, Fei C, Zheng Q, Li X, Chang C. Chidamide, a novel histone deacetylase inhibitor, inhibits the viability of MDS and AML cells by suppressing JAK2/STAT3 signaling. Am J Transl Res. 2016; 8(7): 3169-3178.

3. Jin Y, Lu X Wang M, Zhao X, Xue L. X-linked inhibitor of apoptosis protein accelerates migration by inducing epithelial–mesenchymal transition through TGF-β signaling pathway in esophageal cancer cells. Cell Biosci. 2019; 9 (1): 76. https://doi.org/10.1186/s13578-019-0338-3

4. Kouser L, Paudyal B, Kaur A, Stenbeck G, Jones LA, Abozaid S. M, Stover CM, Flahaut E, Sim RB, Kishore U. Human Properdin Opsonizes Nanoparticles and Triggers a Potent Pro-inflammatory Response by Macrophages without Involving Complement Activation. Front Immunol. 2018; 9. https://doi.org/10.3389/fimmu.2018.00131

5. Ulbrich SE, Rehfeld S, Bauersachs S, Wolf E, Rottmayer R, Hiendleder S, Vermehren M, Sinowatz F, Meyer HHD, Einspanier R. Region-specific expression of nitric oxide synthases in the bovine oviduct during the oestrous cycle and in vitro. J Endocrinol. 2006; 188(2): 205-213. https://doi.org/10.1677/joe.1.06526

6. Aghdaei HA, Kadijani AA, Sorrentino D, Mirzaei A, Shahrokh S, Balaii H, Geraci M, Zali. An increased Bax/Bcl-2 ratio in circulating inflammatory cells predicts primary response to infliximab in inflammatory bowel disease patients. United Eur Gastroenterol J. 2018; 6(7): 1074-1081. https://doi.org/10.1177/2050640618774637

7. Karimi Ardestani S, Tafvizi F, Tajabadi Ebrahimi M. Heat-killed probiotic bacteria induce apoptosis of HT-29 human colon adenocarcinoma cell line via the regulation of Bax/Bcl2 and caspases pathway. Hum Exp Toxicol. 2019; 38(9): 1069-1081. https://doi.org/10.1177/0960327119851255

8. Ofinran O, Bose U, Hay D, Abdul S, Tufatelli C, Khan R. Selection of suitable reference genes for gene expression studies in normal human ovarian tissues, borderline ovarian tumours and ovarian cancer. Mol Med Rep. 2016; 14(6): 5725-5731. https://doi.org/10.3892/mmr.2016.5933
